# From reproducibility to comparability in computational photochemistry: lessons from the PhotoChem explorer prototype

**DOI:** 10.1039/d6fd00102e

**Published:** 2026-07-15

**Authors:** Nanna H. List, Sandra Gómez, Lea M. Ibele, Basile F. E. Curchod, Davide Avagliano

**Affiliations:** a Department of Chemistry, KTH Royal Institute of Technology SE-10044 Stockholm Sweden nalist@kth.se n.h.list@bham.ac.uk; b Science for Life Laboratory Stockholm Sweden; c School of Chemistry, University of Birmingham Birmingham B15 2TT UK; d Departamento de Química, Universidad Autónoma de Madrid Módulo 13 28049 Madrid Spain; e Aix Marseille University, CNRS, ICR 13397 Marseille France; f Centre for Computational Chemistry, School of Chemistry, University of Bristol Bristol BS8 1TS UK; g Chimie ParisTech, PSL University, CNRS, Institute of Chemistry for Life and Health Sciences (iCLeHS, UMR 8060) 75005 Paris France; h IADCHEM, Institute for Advanced Research in Chemistry, Universidad Autónoma de Madrid Madrid Spain

## Abstract

We present the PhotoChem Explorer (PCE) prototype, a proof-of-concept web-based platform that represents computational photochemistry studies of gas-phase molecules in a structured and queryable form. Rather than treating excited-state dynamics simulations as isolated results reported in heterogeneous formats and across different sources, PCE organizes studies according to their molecular systems, computational workflows, methodological choices, and associated observables, facilitating that relationships and trends can be explored systematically across studies. In this way, the prototype illustrates how structured data representations could support improved findability, comparison, reproducibility, and reuse in computational photochemistry. At a fundamental level, it demonstrates how excited-state dynamics studies could become part of a more connected and cumulative body of knowledge, potentially enabling new modes of investigation. The prototype exposes practical challenges that must be addressed to realize this vision, including the development of shared representations, workflow descriptions, and their integration into scientific practice. More broadly, PCE is intended as a practice-driven experimentation platform to stimulate discussion and iterative community development of standards and representations for excited-state dynamics studies.

## Abstract 2

Shared, structured representations can link excited-state dynamics studies into knowledge that is easier to understand, compare, and reuse. PhotoChem Explorer explores what it takes to support more cumulative knowledge building in computational photochemistry.

## Graphical abstract

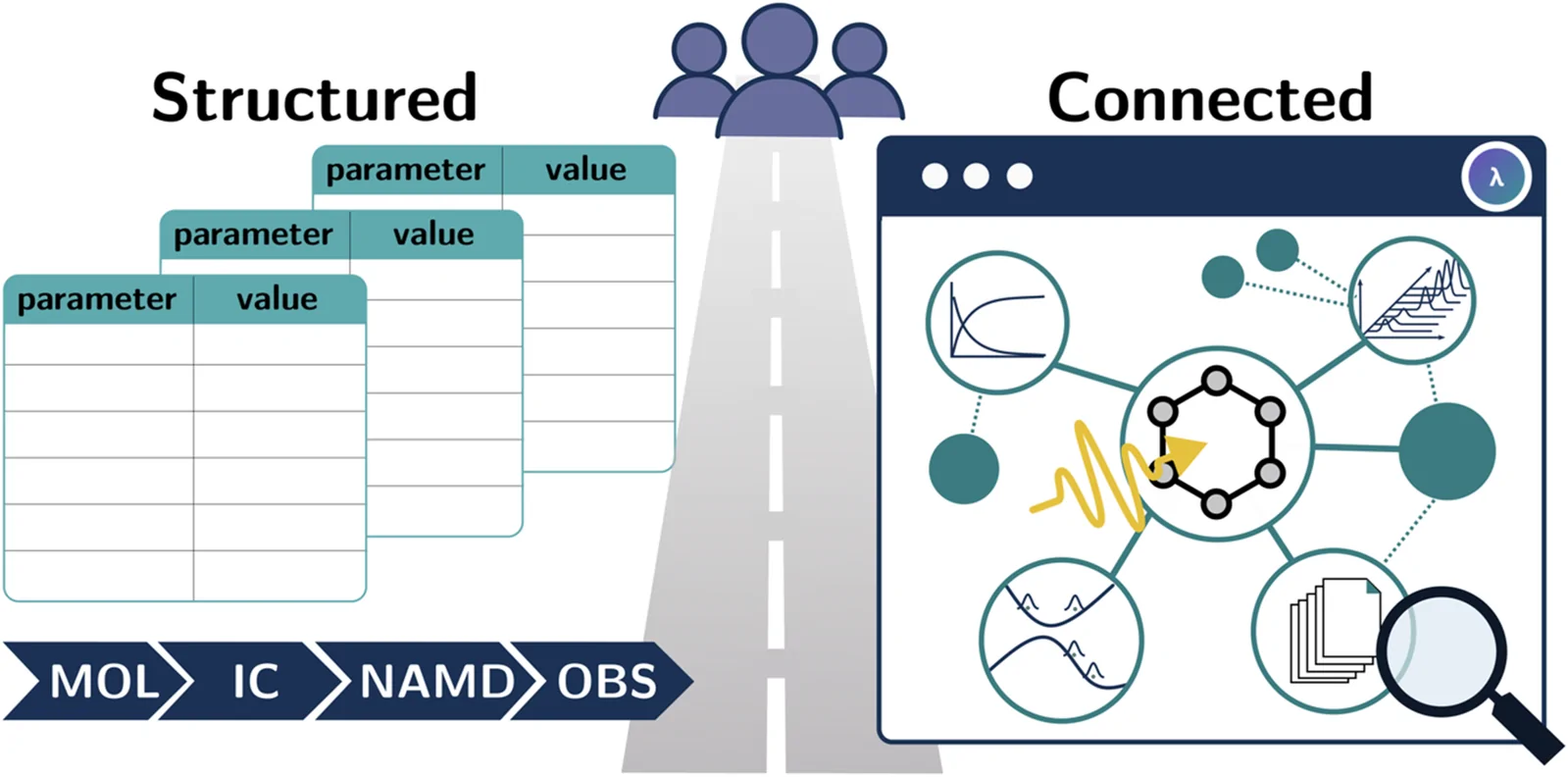

## 1 Introduction

The open science movement^1–5^ and the formulation of the FAIR^6^ (findable, accessible, interoperable, and reusable) principles have contributed to a growing emphasis on the importance of transparency, reproducibility, and sharing of research data and workflows. These developments are increasingly reflected in current practices, as well as in guidelines from research funding agencies and publishers, which promote making research data publicly available, such as through unstructured, general-purpose repositories.^7,8^ In response, more concrete, field-specific efforts are beginning to emerge. For instance, in theoretical and computational chemistry, the *Journal of Chemical Theory and Computation* and the *Journal of Chemical Information and Modeling* very recently published a joint editorial, which introduces a new reproducibility policy, emphasizing the availability of the data, code, and workflows underlying published computational results.^9^ While such developments represent important progress, they primarily address computational reproducibility, *i.e.*, the ability to reproduce results using the same input files, program and program version. However, computational reproducibility alone does not necessarily ensure that workflows are transparent, interpretable, or the results readily reusable beyond the original context. As these practices are adopted, they are likely to result in large collections of heterogeneous files without an explicit representation of the underlying workflows or outputs. As a result, studies may remain difficult to interpret and use in ways that support cumulative knowledge building.

These challenges are particularly acute in computational photochemistry. Photochemistry plays a central role in a wide range of processes, from atmospheric and biological systems to energy conversion and materials science.^10–15^ From an experimental perspective, probing photochemical reactions is challenging, as observed signals often reflect coupled electronic and nuclear dynamics across multiple timescales, leading to overlapping and difficult-to-interpret signatures. As a result, theoretical and computational methods are essential not only for interpreting time-resolved experiments and providing mechanistic insight, but also for guiding experimental design and identifying promising directions for experimental investigation. Describing photochemical processes computationally is, however, equally demanding. Excited-state dynamics simulations are not single-step calculations, but rather involve complex, multi-step workflows that combine initial-condition sampling, electronic-structure methods, non-adiabatic dynamics, and post-processing analysis. Each component requires approximations that must balance accuracy and efficiency, and their interplay ultimately determines the quality of the simulation. Over the past few decades, substantial advances in electronic-structure methods beyond the Franck–Condon (FC) point, non-adiabatic dynamics algorithms, and computer hardware have made such simulations increasingly accessible.^16,17^ This progress is reflected in the growing integration of excited-state dynamics simulations with time-resolved experiments^18–21^ and in the expansion of the field from methodological experts to a broader community of practitioners.^22–24^

**Fig. 1:**
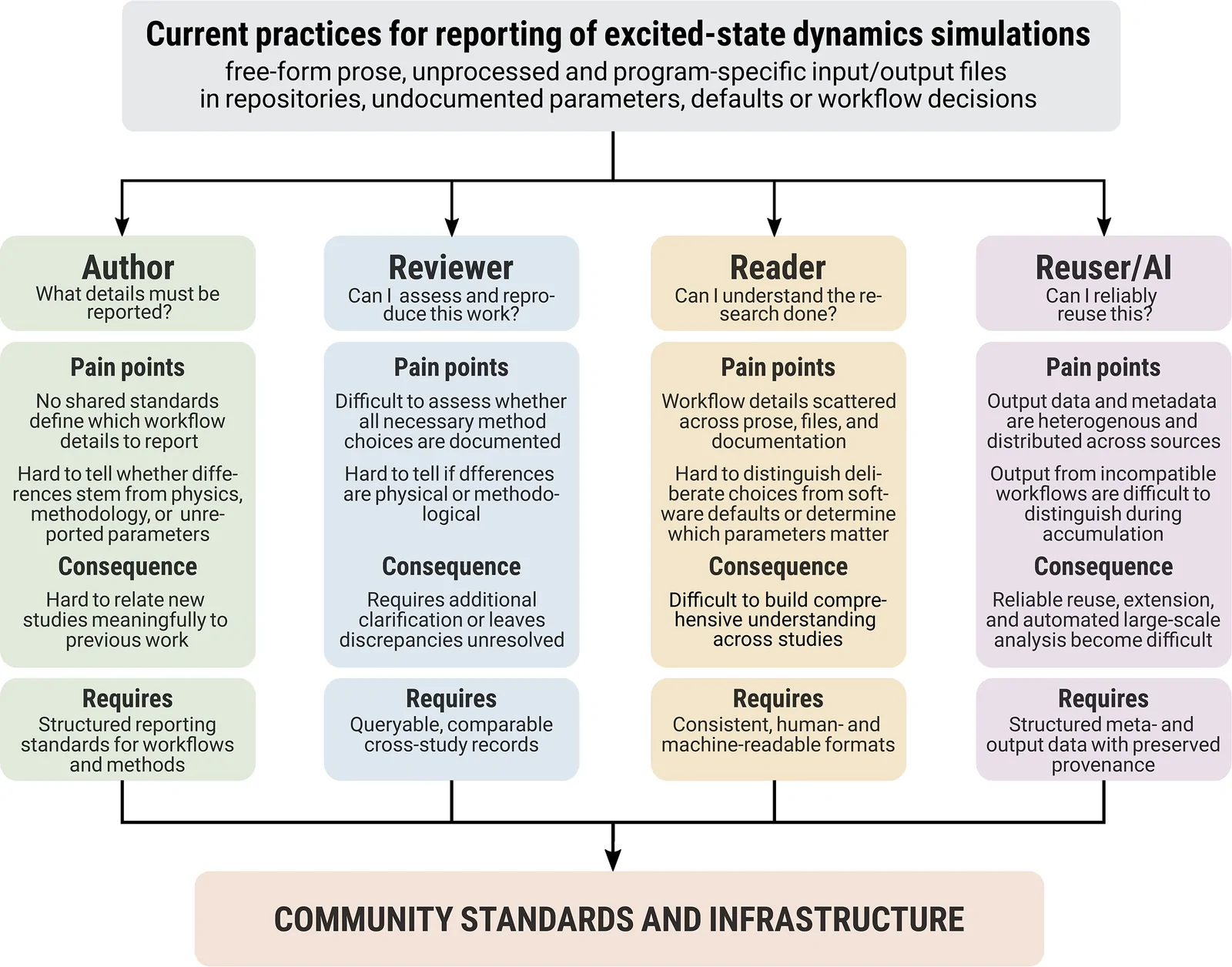
Challenges in reporting, assessing, comparing, and reusing excited-state dynamics studies arising from current reporting practices in the field. Authors, reviewers, readers and reuse face distinct but related difficulties due to the lack of structured and standardized representations of workflows, assumptions, and results.

As a direct consequence of this progress in computational photochemistry, a community-wide prediction challenge^25^ was recently organized, aiming to assess the predictive power of contemporary excited-state dynamics simulations through a double-blind study on the photochemistry of cyclobutanone, with comparison to ultrafast electron diffraction experiments.^26,27^ As summarized in a follow-up perspective,^28^ the challenge gathered fifteen independent theoretical contributions to the *Journal of Chemical Physics* spanning a wide variety of computational approaches. Navigating this landscape naturally involved seeking answers to a range of questions, from mechanistic interpretation and methodological details to predictive accuracy and comparison across studies. Given the comparative nature of the challenge, the manuscripts provided a high level of detail in their description of computational methodologies and parameters. Most contributions reported results through free-form prose, figures/tables, and selected subsets of input/output data, while only a small subset provided more complete input/output in program-specific formats.^29–31^ Nevertheless, comparing the results of the different contributions in a systematic manner proved non-trivial. Understanding how individual results were obtained required interpreting complex, multi-step workflows from textual descriptions, with certain aspects, including post-processing steps and decisions, being sometimes implicit or only partially documented. Consequently, the current reporting practices place a substantial burden across different roles in the research process (Fig. 1). Authors must anticipate and document relevant methodological details, reviewers must assess their sufficiency, readers must reconstruct workflows and interpret decisions from textual descriptions and program-specific setups, and reusers must collect, process, and integrate heterogeneous data across multiple formats and sources.

The critical issues described above are unfortunately not limited to the prediction challenge and reflect the broader conditions in computational photochemistry. While field-agnostic repositories and reproducibility policies represent important progress, they primarily address data availability and methodological reproducibility. What remains largely absent are explicit, interoperable representations of computational workflows, methodological decisions, and generated results that support discoverability, transparent interpretation, systematic comparison, and reuse across studies. In computational photochemistry, coordinated efforts are beginning to emerge, including initiatives to unify non-adiabatic dynamics methods within common software frameworks (*e.g.*, the COSMOS project^32^ around Quantics,^33^ as well as the Newton-X^34^ and Libra^35^ packages) and community benchmarking and calibration studies.^17,28^ Structural biology provides a leading example of what is possible: community standards, curated data, and a coordinated ecosystem of public repositories for raw and processed data, such as the Protein Data Bank (PDB),^36–38^ has enabled large-scale aggregation, cross-study comparison, and reuse of macromolecular structure data. This infrastructure has, in turn, enabled new computational methods and analyses that would have otherwise been out of reach.^39^

In this work, we explore how structured representations of excited-state dynamics simulations can support improved findability, reproducibility, comparison, and reuse across computational photochemistry studies. For this purpose, we developed the PhotoChem Explorer (PCE) prototype as a practical test case to examine how real computational photochemistry studies can be translated into structured and queryable representations. The prototype is intended both as an initial implementation of this idea and as an experimental environment for identifying what information needs to be represented, how entries should be checked and updated, and what community processes would be needed for such an approach to develop into a mature platform.

## 2 The PhotoChem explorer initiative

**Fig. 2:**
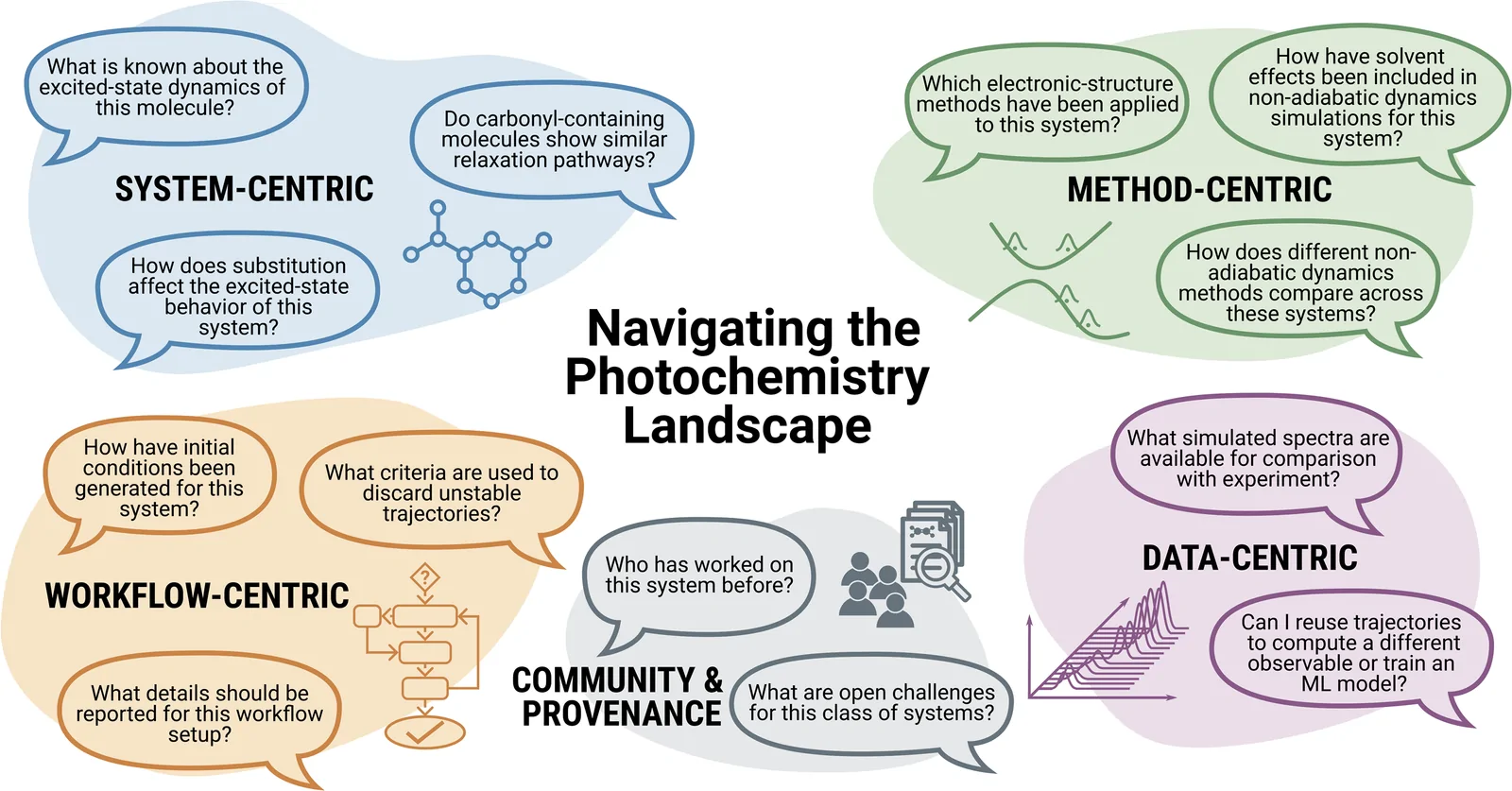
Examples of research questions, organized by query perspective, that the PhotoChem explorer initiative aims to enable.

Imagine a setting where excited-state dynamics studies can be explored not as individual results, but as part of an evolving community platform, built upon structured and queryable representations of computational workflows and results. In such an environment, questions about how outcomes depend on molecular systems, computational methods, and modeling choices could be posed and answered systematically across the literature. Fig. 2 illustrates how this fundamentally different mode of navigating the computational photochemistry landscape could open new possibilities for exploring relationships, trends, assumptions, and discrepancies across systems, methods, and observables, enabling accumulation and reliable reuse of data, and supporting broader community questions such as benchmarking and roadmapping of future methodological developments and experiments. At the same time, this ideal setting could support the full research lifecycle, serving authors documenting workflows, reviewers in assessing their completeness, readers in interpreting results, and reusers in accessing and analyzing the underlying data. Realizing this vision requires representing simulations in a structured and comparable way, such that underlying assumptions, methodological choices, and outputs are defined and can be related across studies, rather than remaining embedded in prose descriptions, supplementary information, and heterogeneous collections of input and output files. The particular interface through which such information is accessed may evolve, but the underlying requirement remains that such details are made explicit enough to be inspected, compared, and reused. In the following, we present the PhotoChem Explorer (PCE) prototype (https://photochem-explorer.com) as a proof-of-concept web-based platform for exploring structured representations of computational photochemistry studies. The prototype is intended as an experimental environment in which such representations can evolve iteratively through practical use and community feedback. In the present form, the platform uses structured metadata to test how improved findability, reproducibility, and comparability could be supported for excited-state dynamics studies. The details of its implementation are given in Section Implementation of the PCE prototype.

### 2.1 The PhotoChem explorer prototype

At the core of the PCE prototype, we represent individual studies in a structured and comparable form. Rather than treating results as isolated outputs, we organize studies according to their underlying computational workflows and associated methodological choices, providing a consistent basis for comparison and exploration across studies. Within the platform, each entry corresponds to a computational photochemistry study: a published investigation of a molecular system together with the specific computational workflow used to obtain the reported results. This includes initial-condition generation (IC), non-adiabatic dynamics (NAMD), and subsequent analysis or observable calculations (OBS), along with the associated methodological choices. At present, the studies included in the prototype are not intended to provide representative coverage of the field, but serve as test cases for developing and evaluating structured representations drawn from the authors' own work. They cover three common non-adiabatic dynamics methods: trajectory surface hopping (TSH^40^), *ab initio* multiple spawning (AIMS^41^), and direct-dynamics variational multiconfigurational Gaussian (DD-vMCG^42^) dynamics. This choice reflects both the exploratory nature of the prototype and the need for detailed knowledge of the underlying workflows and methodological decisions in order to define structured representations. The test cases allow us to examine what information can be represented reliably and what standardization challenges arise when real excited-state dynamics studies are translated into structured form. This process also highlights a broader challenge: the level of detail in current reporting is often insufficient to support fully structured and comparable representations. In this initial phase, we therefore relied on studies where we could directly inspect and revisit the underlying workflows and inputs.

#### 2.1.1 Navigating the photochemistry landscape

From a user perspective, the platform is designed to support a set of common tasks in excited-state dynamics research: identifying relevant studies, understanding how they were performed, and ultimately comparing their results. These tasks correspond directly to the types of questions outlined in Fig. 2, and form the basis for how the platform is structured and navigated.

**Fig. 3:**
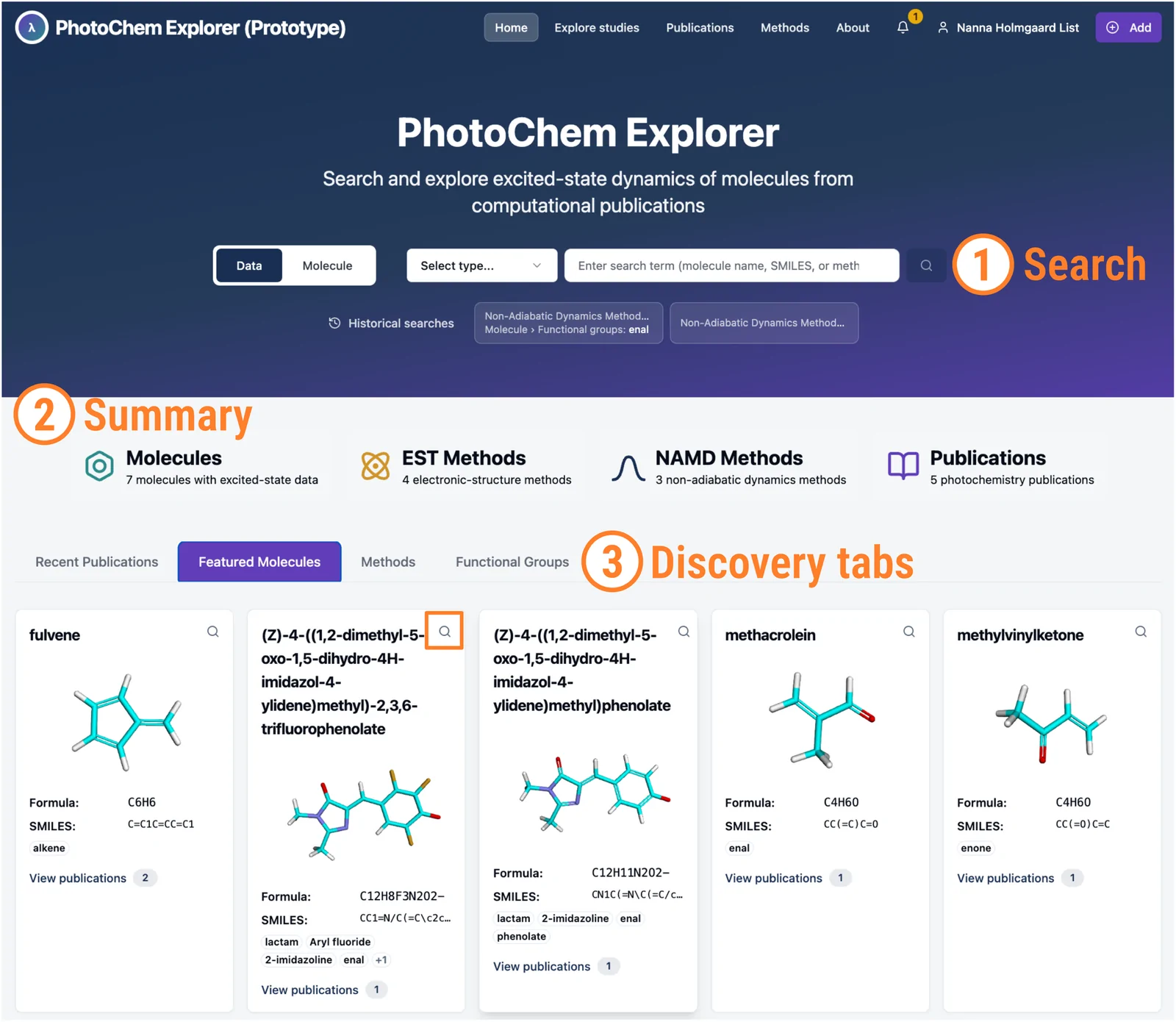
Front page of the PCE prototype. ① The search interface provides an entry point for querying the database by molecule or computational metadata. ② The summary panel provides an overview of the key statistics of the available data. ③ The discovery tabs highlight featured molecules, methods, and publications to support exploration. The magnifying glass in the molecule cards gives an overview of the available studies for the given system.

**Fig. 4:**
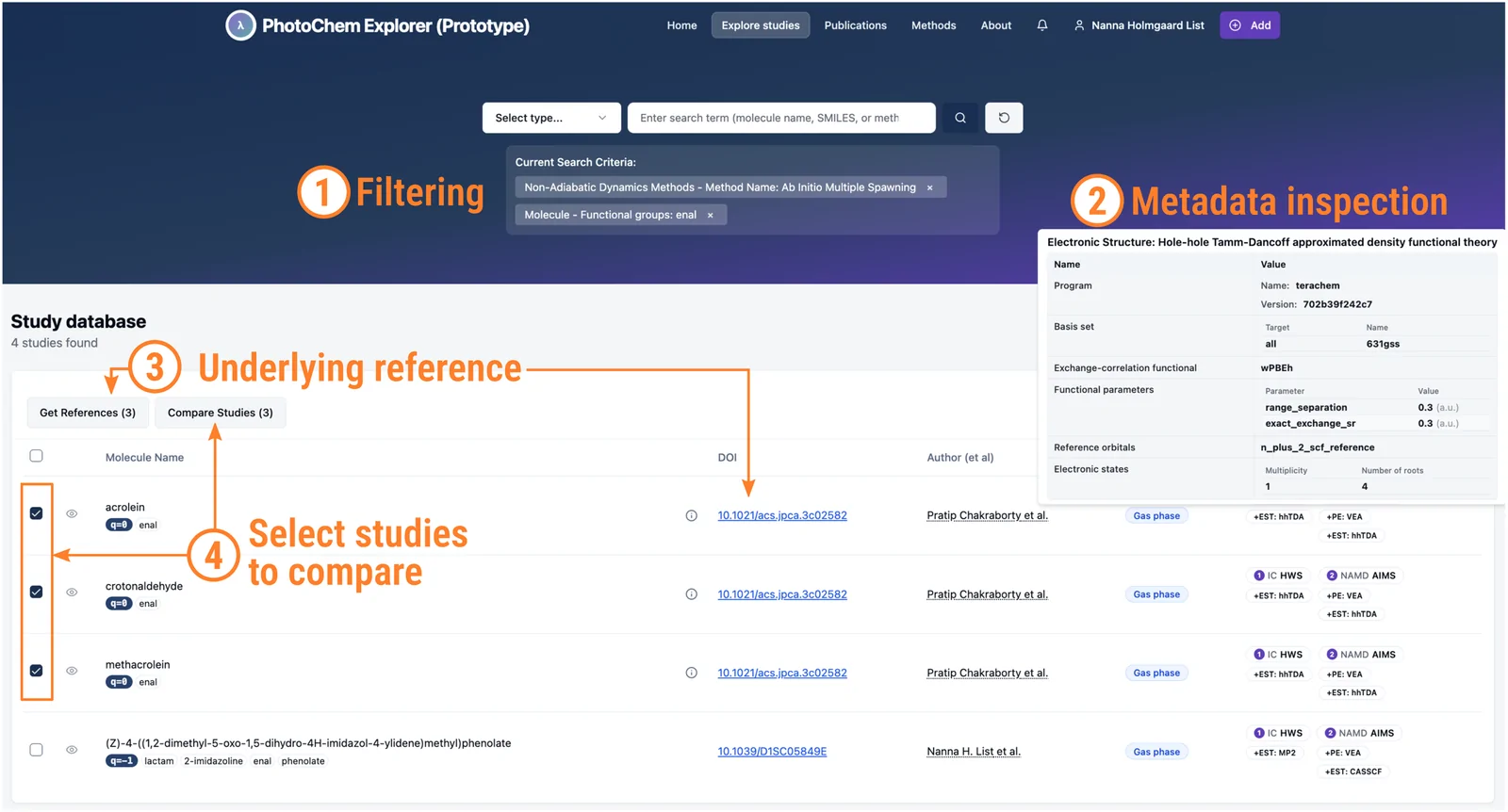
Explorer view showing search results after applying filtering. ① Search criteria allow for filtering the database across the molecular system, workflow steps and methods. ② The metadata associated with each study can be directly inspected, and ③ the underlying publication and its reference accessed. ④ Studies can be selected and compared side-by-side as shown in Fig. 5.

**Fig. 5:**
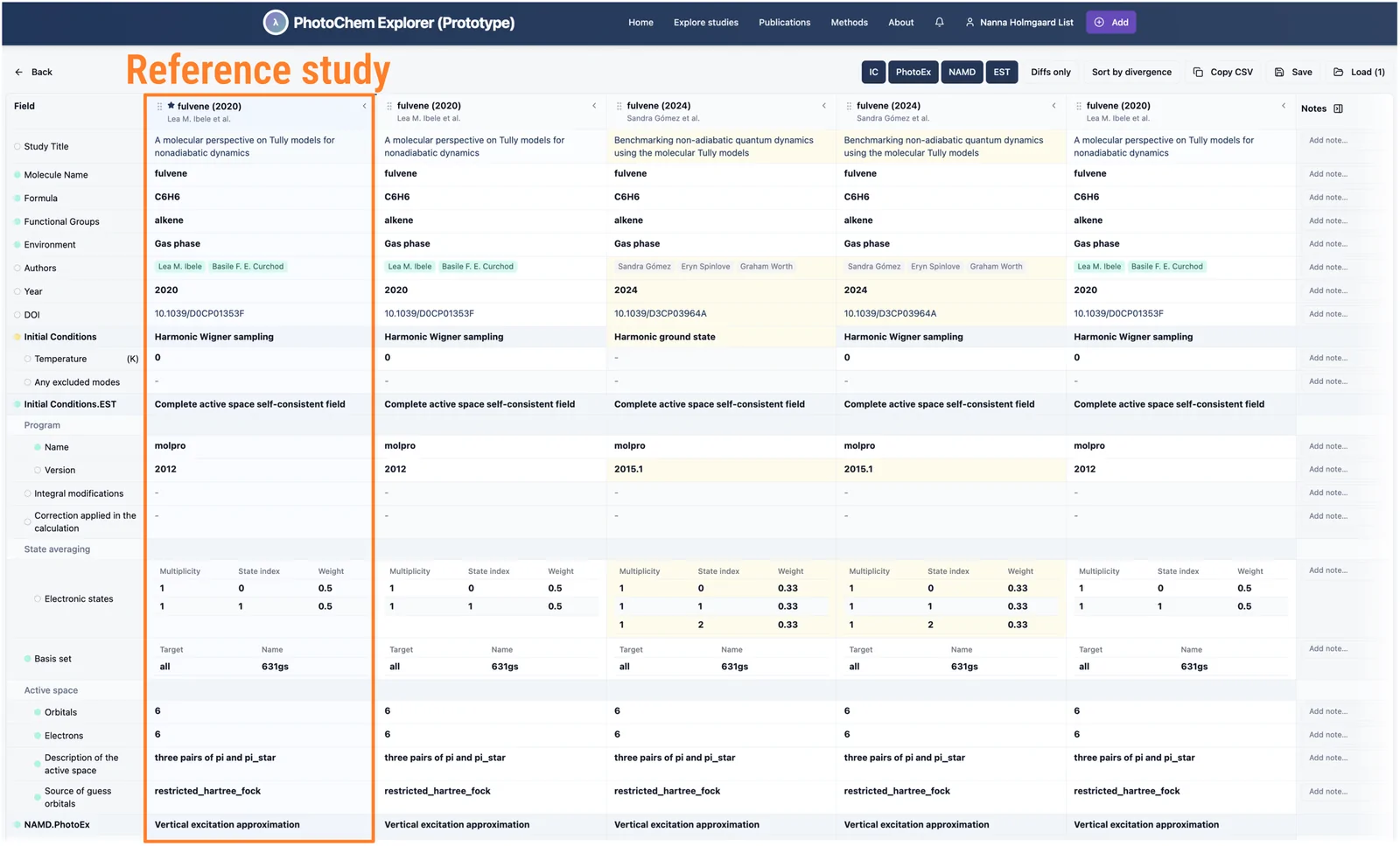
Side-by-side comparison of metadata for selected studies. Workflow components and associated metadata fields are aligned across studies, with visual cues indicating differences to the reference (first-listed) study. The underlying representations make reporting requirements explicit, illustrating how structured metadata can promote more complete and consistent documentation of simulation workflows.

• Search and discovery: as researchers, we typically begin by identifying studies relevant to a given system, method, or research question. The front page serves as the entry point for this process, supporting both targeted searches and discovery (Fig. 3). Studies can be queried using domain-specific attributes such as molecular systems, computational methods, or publication metadata, reflecting how questions are posed in practice. In addition, a discovery layer highlights elements such as molecules, methods, and publications, offering starting points for exploration and illustrating which aspects of studies are structured and searchable within the platform.

• Filtering and inspection: once an initial set of relevant studies has been identified, the selection can be inspected and further refined. The explorer view (Fig. 4) presents all matching studies and allows iterative filtering across system, method, and workflow dimensions. Each study is summarized through key attributes, while detailed metadata can be accessed to examine the simulation workflow, including methodological choices. This makes it possible to understand how individual studies were constructed beyond what is typically accessible from publication summaries alone. Fig. 4 shows an example, where the database has been filtered based on studies of enal-containing systems that employ AIMS. Detailed metadata for each study can be accessed directly from the table, for example by inspecting individual attributes *via* the hover tooltip. From this view, it is also possible to access the associated publications and retrieve the corresponding citation information.

• Comparison: selected studies can also be compared side-by-side. The comparison view (Fig. 5) will maximally align workflow components and associated metadata fields, allowing for inspection of similarities and differences. In practice, comparing studies in the literature requires manually extracting workflow details across papers and attempting to reconcile differences from the reporting. The prototype makes this process straightforward and explicit such that differences are highlighted using simple visual encoding. The view further emphasizes the most informative distinctions by prioritizing fields where studies diverge, allowing attention to focus on the aspects that are most likely to affect the outcome. The example shown in Fig. 5 compares metadata from excited-state dynamics studies on fulvene available in the PCE database.

#### 2.1.2 Connecting your study to the platform

Beyond exploring existing studies, the platform also encourages sharing new ones. Contributing a study implies constructing a new database entry that makes explicit how a given excited-state dynamics study was carried out in terms of the molecular system, simulation workflow, and associated methodological choices. As discussed above, existing studies often report such details with varying levels of completeness and using non-standardized terminology, which can make it difficult to reconstruct workflows or compare results directly. A structured representation addresses this by making these elements explicit and consistently defined, enabling a more complete account of how results are obtained and supporting comparison across studies. In the prototype, this is achieved by providing a step-by-step form to input metadata based on workflow steps and methods chosen. The fields either offer predefined types or options to ensure a consistent vocabulary, necessary for meaningful search and comparison. This shifts reporting from free-form descriptions to structured representations. Although the platform does not yet capture output data directly, submissions can include links to external repositories, providing visibility of underlying input and output files.

#### 2.1.3 Maintaining a shared resource: roles, review and versioning

Since the prototype is intended as a shared and community-editable resource, it naturally brings the need for some level of curation, including a review and stewardship process. Contributions can include both new studies and refinements of existing as well as suggestions to new methods or workflow components. To support provenance, changes are versioned, allowing the evolution of methods to be tracked and enabling users to inspect how entries have been modified. While all curated studies can be freely explored, contributions are restricted to registered users, and submitted entries are reviewed by moderators to ensure compliance with the defined metadata standards. Such standards, introduced here for a small set of methods as part of the prototype, are intended to be defined, refined and expanded through community discussion and use. In this sense, the platform aims to foster community ownership of how general data management principles are translated into concrete, shared practices in computational photochemistry.

#### 2.1.4 How studies are represented and connected

The prototype features described above rely on representing excited-state dynamics studies in a structured and consistent way. This is achieved through an underlying data model for computational photochemistry metadata, which defines how the key elements of a study are described and related. Conceptually, a study connects a publication, a molecular system, and a computational workflow composed of one or more components (*e.g.*, IC, NAMD, OBS) in a so-called relational database. Each workflow component is associated with one or more methods and their method-specific parameters. Allowing for multiple methods within each workflow component is necessary because individual steps are often defined by combinations of methodological choices rather than a single method. For example, in the NAMD component, it is generally not sufficient to detail only the dynamics scheme (*e.g.*, trajectory surface hopping, TSH) but also requires specifying the electronic-structure method used (*e.g.*, CASSCF). Method definitions are stored once and referenced wherever they are used, enabling queries such as “which molecular systems has this method been applied to?” or “how do parameter choices differ across studies using the same method?” To accommodate future revisions of methods, we use a versioned and flexible (key-value data) format, such that any additions or changes will not break backward compatibility but can rather be added as a new version of the parent method. The same principle applies to molecules, as they can have distinct variants, such as different charge states, stereoisomers, or protonation states, each is represented as a separate molecular entry linked to the relevant publication and metadata, making them individually searchable while preserving the specificity needed for meaningful comparison.

## 3 Challenges revealed by the prototype

Building and populating the prototype with real studies exposed a number of challenges related to metadata formalization, workflow representation, and community adoption. These observations arose directly from the practical task of structuring existing studies and point to considerations that extend beyond any single platform. In the following, we discuss these challenges and the insights they provide.

### 3.1 Metadata standardization is inherently a community question

As we worked to define structured metadata representations, we quickly realized that metadata standardization involves not a single design decision, but a set of interrelated ones. Beyond defining an overall data model (publication, molecule, workflow components, methods), it was necessary to determine which attributes are shared across methods, what level of detail is sufficient to describe a method (*e.g.*, parameters, value types, and constraints), and how this information should be expressed in a consistent way (terminology and naming conventions). Even within the small set of studies used as test cases in the prototype, these questions proved challenging and required detailed knowledge. This experience highlighted that meaningful metadata standards cannot be defined in isolation, but will require coordinated input from experts across both non-adiabatic dynamics and electronic-structure methods. An additional challenge is ensuring consistency across methods. While different methods require distinct parameters, comparable aspects must be represented in a consistent way to enable cross-study queries and comparisons.

### 3.2 Practical limits to general representations

The prototype was developed around gas-phase molecular systems, where the molecule itself serves as the primary object of study. This setting allows us to leverage established cheminformatics concepts and tools, including molecular identifiers, structure representations, and automated annotation of functional groups. In particular, the prototype currently incorporates both the Simplified Molecular Input Line Entry Specification (SMILES^43^) and, where available, the IUPAC International Chemical Identifier (InChI ^44^) as searchable molecular representations. While these representations are established and suitable for many molecular systems, they are not universally applicable. More importantly, extending the prototype beyond relatively simple gas-phase molecules introduces additional challenges in defining and representing the relevant photochemical system. For example, in the case of a solvated chromophore, it becomes less clear how to define the system and its representation. Unlike isolated molecules, such systems generally lack unique identifiers, and different parts of the system are often described at different theoretical levels. The chromophore may be treated quantum mechanically, whereas the environment may be represented implicitly through a continuum model, explicitly using molecular mechanics force fields, or a lower-cost electronic-structure method. Representing such systems therefore requires additional layers of metadata to describe how different parts of the system are modeled and coupled. The challenge becomes even more pronounced in multichromophoric assemblies, and molecular materials, where the distinction between chromophore and environment may itself become ambiguous or physically less meaningful. Extending the framework toward such applications will therefore require more flexible abstractions of what constitutes a “system”, while still preserving the ability to meaningfully compare studies.

A related challenge concerns the level of generality used to represent simulation workflows. The prototype currently supports linear workflows. While it would be desirable to accommodate workflows of higher complexity, the increased flexibility introduces practical limitations. More complex workflows become progressively harder to visualize, interpret, and compare consistently, while the effort required to describe them also increases. This highlights a fundamental tension between generality and usability: representations capable of capturing all possible workflow variations may ultimately become difficult to use in practice, both for data entry and for comparative analysis.

### 3.3 Tension between existing practices and structured representations

Applying the framework to the selected test studies revealed that current reporting practices often do not fully capture the proposed metadata structure, resulting in incomplete or missing fields. This observation highlights a central tension in determining how to incorporate existing studies while progressing toward forward-looking metadata standards. Allowing previous studies to be included, even if they do not fully satisfy the metadata framework, would increase coverage and make the platform more immediately useful. However, such an inclusion may introduce inconsistencies and limit comparability across studies. Conversely, restricting contributions to studies that comply with agreed-upon standards would promote consistency and clarity, but at the cost of excluding a large portion of the existing literature, particularly during early adoption. In practice, intermediate approaches may be required, such as explicitly annotating studies with their relationship to metadata standards (*e.g.*, predating its introduction), as well as associating entries with evolving representations versions. The extent to which existing studies are incorporated would ultimately depend on community engagement and curation efforts, such as automated metadata extraction from the literature.

### 3.4 Decision and evaluation concepts are underdeveloped

Excited-state dynamics simulations rely on multiple methodological decisions that can have important downstream consequences,^24^ yet these are currently not described in a systematic way. In particular, evaluation criteria (*e.g.*, stability and trajectory survival) are often only implicitly defined, making it difficult to reconstruct both the workflow itself and the scientific reasoning underlying specific methodological choices. More fundamentally, this is not only a question of missing metadata, but of missing conceptual clarity. There is currently no shared framework for how such decisions, assumptions, evaluation steps, and robustness considerations should be described and consistently referred to. As a result, these aspects are often reported in an ad hoc manner, if at all. This is problematic because methodological decisions are shaped by practical experience, failed attempts, or known limitations that remain largely invisible in the published literature. Consequently, it may be unclear why a particular choice was made, how robust it is, or what alternative approaches were explored and discarded. For example, the four-electron, three-orbital active space previously used in active-space-based dynamics simulations of the anionic green fluorescent protein chromophore^45,46^ exhibits some instability upon twisting around the phenolate bond due to orbital rotation between the active and inactive spaces. Such information is typically difficult to represent systematically, despite potentially being highly valuable for future studies and method development. More generally, the field currently lacks mechanisms for capturing negative methodological knowledge, such as parameter choices, basis sets, or electronic-state selections that were found to produce unstable or qualitatively poor behavior. Structured representations of such considerations could help build more cumulative understanding of methodological robustness and unresolved limitations across systems and methods. In the prototype, we made initial attempts to capture some of these elements through a semi-structured approach combining common keywords with free-text descriptions within the specifications of workflow components. However, these attempts made it clear that meaningful standardization in this area will require the community to first articulate and agree on the underlying concepts themselves.

### 3.5 Adoption depends on integration with publication and review practices

Another challenge revealed by the prototype is that even well-designed metadata structures will not be widely adopted unless they become embedded in scientific practice. This will likely require changes to how studies are prepared, reported, and reviewed, such that structured metadata becomes an integrated component of the research workflow rather than an additional reporting burden. Although the prototype provides structured templates for metadata reporting during manuscript preparation, it currently functions as an after-the-fact reporting tool. As a result, the prototype represents only a partial integration, as metadata entry remains separate from publication and review workflows. This separation effectively introduces additional work on top of existing reporting requirements, without an immediate pay-off for authors. Addressing this issue will require the development of mechanisms that integrate structured metadata reporting into existing publication and review processes.

### 3.6 Reuse requires standardization of output data

While structured metadata supports discovery and comparison across studies, cumulative knowledge building and reliable reuse in computational photochemistry depend on shared practices for representing, reporting and validating simulation outputs. This challenge appears at both the level of data interoperability and the level of methodological evaluation. First, simulation outputs that may serve as input for subsequent calculations would benefit from standardized representations to facilitate downstream analysis and reuse, for example in machine learning applications or additional observable simulations. Second, computational photochemistry currently lacks shared benchmarking practices, such as consensus on minimal benchmark quantities and validation tests to report. While the performance of many electronic-structure methods is well-characterized near the FC region, far less benchmark data exist across the broader configurational space important to photochemical processes. As a result, benchmark results from different studies are often difficult to compare and combine into a cumulative body of knowledge, and the benchmarks most informative for assessing the reliability of excited-state simulations are still to be established. This was evident in the recent prediction challenge, where different studies reported different benchmark quantities and no clear relationship emerged between the benchmark performance and excited-state dynamics outcomes.^28^ These observations suggest that simulation outputs, benchmark quantities and validation practices represent important targets for further community-level canonicalization.

## 4 From prototype toward community standards and infrastructure

The previous section highlighted some practical challenges associated with standardization, workflow representation and community adoption. In this section, we discuss possible paths forward to address these challenges. As in the prototype, we limit our focus to gas-phase molecular systems, where substantial progress can already be made before extending these approaches to more complex photochemical systems.

### 4.1 Defining and implementing standardized representations

A central challenge is the definition and implementation of standardized representations. Based on our experience with the PCE prototype, we argue that this challenge is best approached iteratively through practical use and community refinement, rather than through a fully specified standard defined upfront. The standardization task spans multiple levels:

• Method metadata: to map out which aspects of methods should be reported and how they can be meaningfully standardized, a practical starting point is to examine how the same method is represented across different software implementations. This mapping allows identification of parameters whose meaning and role are sufficiently aligned to support shared schema elements and consistent naming conventions for structured method representations. At the same time, non-overlapping parameters must be retained to preserve method- and implementation-specific details, while still being represented in a structured form that supports interpretation and future comparison. A related challenge concerns identifying common concepts across different methods within the same workflow category. Here, intersecting fields and higher-level abstractions can provide the basis for comparison across methods, provided that sufficiently consistent interpretation and terminology can be established. This process extends beyond field names to include agreement on value types, units, and controlled vocabularies, forming the basis for more interoperable, searchable, and comparable workflow representations. Such representations also help clarify which aspects of workflows are expected to be reported, thereby supporting more consistent and reproducible documentation of excited-state dynamics studies.

• Decisions and evaluation criteria: capturing decision and evaluation criteria presents a particular challenge, as it reflects elements of the underlying scientific reasoning. A practical next step is therefore to develop lightweight representations for documenting workflow decisions, filtering procedures, and evaluation criteria in a more systematic way. Building on the initial attempts in the prototype, one possible approach is to combine controlled keywords with free-text descriptions that can gradually be refined into more structured representations through community use and comparison across studies. Particular attention should be given to workflow evaluation and trajectory filtering procedures, where criteria for discarding trajectories or assessing simulation stability are often applied implicitly and reported inconsistently across studies. Explicitly representing such decisions in a structured form would improve transparency and reproducibility while also helping to identify recurring practices and robustness considerations across methods and systems.

• Output data: output can be divided into (at least) two categories: (1) raw excited-state dynamics simulation data; and (2) post-processed data, such as the output from additional observable calculations. The former can serve as input for further calculations, including simulation extensions, post-processing or training of machine-learning models, while the latter is primarily used for analysis, such as comparing predictions across methods or to experiments. Standardizing such outputs involves defining data formats, consistent conventions for units, and the meaning of reported quantities. As raw simulation data forms the foundation for downstream calculations and reuse, it is natural to prioritize standardization efforts at this level. For trajectory-based methods, initial steps in this direction have already been taken. For example, SHNITSEL (Surface Hopping Nested Instances Training Set for Excited States) introduces a structured representation in binary NetCDF-4 (ref. 47) format for TSH datasets, and organizes both new and previously reported Wigner-based initial conditions together with selected output from TSH simulations of nine organic molecules.^48,49^ The SHNITSEL-data datasets are distributed through a general-purpose Zenodo repository and have been reused in machine-learning applications.^50^ The SHNITSEL-tools, an associated Python package for processing and analyzing SHNITSEL-formatted output, further demonstrate how shared output representations can support automated analysis, filtering, visualization, and comparative exploration of excited-state dynamics simulations across molecular systems.^51^ Similar principles could be extended to other trajectory-based methods, such as AIMS and DD-vMCG, by incorporating the additional quantities and representational flexibility required to describe evolving nuclear wavepackets. More broadly, SHNITSEL illustrates that meaningful progress toward standardized output representations can already be achieved through shared formats and conventions without dedicated domain-specific database infrastructure.

• Minimal benchmark sets and validation measures: a further step toward comparability is the definition of a minimal set of benchmark outputs that should be reported across studies. The goal is not to establish a complete benchmarking protocol, but to ensure that each study produces a small set of consistently defined quantities that can be accumulated and directly compared over time. Existing community efforts, such as the *Best Practices for NAMD Simulations*^24^ living journal and the *Molecular Benchmarks in Non-Adiabatic Dynamics*^17^ initiative, provide natural starting points for identifying candidate quantities. For example, to assess electronic-structure quality beyond the FC point, one could report minimum-energy paths between the FC geometry and relevant excited-state minima and conical intersections, using an agreed-upon protocol. Defining such quantities in a consistent way would enable direct comparison across studies and support cumulative knowledge building beyond the FC region. In this way, benchmarking would become an integrated part of the literature, rather than being limited to dedicated benchmark studies.

Community workshops could provide a platform to discuss and develop method and output representations. To enable productive discussion, such efforts would likely benefit from research groups preparing and submitting candidate representations in advance, providing a structured basis for comparison and refinement. These settings could support focused discussions within working groups, with outcomes shared and iteratively improved. In this context, the PCE prototype can serve as an experimentation platform, allowing proposed representations to be implemented, tested, and refined. Methodologically diverse studies, such as those from the cyclobutanone prediction challenge, provide a useful testbed for evaluating candidate representations, although additional cases would be required to capture the diversity of systems. More focused follow-up efforts could then support the gradual standardization of these representations through application across a broader range of studies.

### 4.2 Integrating metadata generation and output standards into simulation workflows

A key step toward scalable standardization is to integrate the generation of structured metadata and output representations directly into simulation workflows. Instead of relying on post-hoc reconstruction from program-specific files, simulation codes and workflow managers should be able to produce structured representations of both inputs and outputs in accordance with shared definitions. There are already initial examples of this practice. The Newton-X package provides program-specific utilities that automate the extraction and organization of simulation data through post-processing of input and output files into compressed trajectory data and descriptive summaries.^34^ The SHNITSEL-tools introduces parsers that translate TSH outputs from established NAMD packages, such as Newton-X^34^ and SHARC,^52^ into a common format. In the context of PCE, this parser-based strategy provides a practical near-term route: program-specific parsers could transform existing simulation inputs and outputs into structured metadata (and ultimately data) representations. For this to be effective, simulation packages would need to expose sufficiently complete workflow information, including both user-defined parameters and program defaults. But community-level standardization could also support a broader shift in practice, where structured metadata and output representations are generated directly within simulation software and workflow tools, alongside native program-specific files. Such an approach would reduce the need for downstream reconstruction and facilitate direct integration into shared platforms and repositories. Some degree of post-processing will nevertheless remain necessary since excited-state dynamics simulations often rely on multiple trajectories that must be analyzed collectively to reconstruct the evolving molecular wavepacket. Importantly, however, standardization need not constrain methodological innovation. Provided that representations remain extensible, new methods and workflow developments can be incorporated without breaking compatibility, allowing standardization and methodological development to evolve in parallel.

### 4.3 Integrating structured metadata into publication and review practices

One possible mechanism to support adoption of structured metadata representation is the introduction of persistent unique identifiers, analogous to PDB identifiers in structural biology. In the context of the PCE prototype, such identifiers could be assigned to individual recorded studies and referenced directly in data availability statements, providing a visible marker that a study has been documented in a structured and reusable form. The use of such identifiers also creates a concrete incentive structure: authors gain visibility and recognition for following community standards, while reviewers are given a simple and low-burden mechanism for assessing and encouraging reproducibility, for example by checking for the presence of an identifier. Over time, such mechanisms could help make structured workflow representations a more visible and expected component of computational photochemistry studies.

### 4.4 Building forward while incorporating past studies

The tension described in Section 3.3 suggests that population should not be treated as a single uniform process. While the platform is forward-looking in the sense that new studies can be shaped by emerging reporting standards, it should also allow studies from the existing literature to be incorporated. A practical strategy for accommodating both routes would be to make the status of each entry explicit. For example, records could indicate whether the metadata were supplied by the original authors, reconstructed from the publication and supporting files assisted by automated extraction, or entered before a given standard was introduced. Such annotations would allow studies from the existing literature to be included without implying that all entries support the same level of details.

### 4.5 Establishing processes for maintaining and evolving standards

While initial standardization efforts can emerge through focused community initiatives, maintaining and evolving such representations will ultimately require some level of sustained coordination. At the present stage, however, it is likely more important to establish lightweight and flexible mechanisms for community interaction than fully developed governance structures. One possible direction is the formation of focused working groups that periodically discuss and refine emerging representations, drawing inspiration from related efforts, such as the recent initiatives in classical molecular dynamics.^53–56^ In this context, living “good-practices” articles could provide a flexible mechanism for documenting evolving recommendations, conventions, and benchmark quantities as community experience accumulates. Longer-term sustainability of such efforts, including questions of infrastructure hosting, curation, validation, and community stewardship, will likely require dedicated organizational and funding models beyond the scope of the present prototype. Lightweight and community-driven mechanisms would nevertheless allow standards and representations to evolve iteratively through continued methodological developments, community discussion, and practical application across studies.

## 5 Conclusion and outlook

The PhotoChem Explorer (PCE) prototype was developed as a practical experiment in representing excited-state dynamics studies as structured, queryable entries rather than only as isolated textual reports. Through the process of implementing and populating the prototype with a small test set of real studies, it became clear that the central challenge is not only technical, but also conceptual: excited-state dynamics simulations involve complex workflows, methodological assumptions, evaluation criteria, and representation choices that are essential for interpretation, yet are rarely described in a systematic form.

More broadly, the prototype illustrates how structured representations could support a more cumulative and queryable body of computational photochemistry knowledge, where workflows, methodological decisions, and observables become systematically accessible and comparable across studies. In this sense, the work points towards new possibilities for reproducibility, comparison, reuse, and large-scale analysis that are currently difficult to realize when studies are primarily communicated through heterogeneous formats, textual descriptions and figures.

At the same time, the experience with the prototype highlights that improving reproducibility and comparability will require more than the definition of metadata schemas alone. Shared representations must emerge across multiple levels, including method metadata, workflow descriptions, evaluation procedures, benchmark quantities, and output data. Some areas, particularly workflow decisions, robustness evaluation, and negative methodological knowledge, currently lack shared conceptual frameworks. Nevertheless, the prototype also suggests that useful progress does not depend on fully developed standards from the outset. Even relatively lightweight forms of structured reporting, such as workflow metadata tables (in addition to the usual free-form text) and more consistent descriptions of methodological procedures, could already make studies easier to understand, relate across the literature, and process with automated tools.

A further lesson from the prototype is that standardization must become integrated into scientific practice in order to scale. Structured metadata and output representations should ideally be generated directly within simulation workflows and connected naturally to publication, review, and data-sharing processes. In the longer term, one can envision structured workflow metadata and output data becoming linked to publications through persistent identifiers referenced directly in data availability statements, helping to make reproducibility and reuse more visible components of research dissemination. As AI-based approaches make it easier to extract and reuse information at scale, such links would also make the provenance, completeness, and consistency of the underlying information easier to assess, supporting more reliable reuse.

Developing shared, structured representations and supporting tools and practices for excited-state dynamics will benefit from interactions between multiple complementary initiatives addressing different aspects of the problem. These include efforts toward shared software environments spanning multiple non-adiabatic dynamics methods, output representations, parser and interoperability tooling, benchmark initiatives, and evolving community best-practices recommendations. In the long term, such developments could help shift computational photochemistry toward a more connected and cumulative mode of research, in which studies can be systematically related across systems, methods, workflows, and observables, and where new work builds more directly on structured knowledge and data derived from previous simulations. In this context, we hope that the PCE prototype can serve as an experimentation environment for developing, testing, and refining shared representations and practices through future community engagement and practical application.

## Author contributions

N. H. L. conceptualization (lead), software (lead), data curation (equal), formal analysis (lead), visualization (lead), project administration (lead), writing – original draft (lead), writing – review and editing (equal); S. G. conceptualization (supporting), methodology (equal), data curation (equal), writing – original draft (supporting), writing – review and editing (equal); L. M. I. conceptualization (supporting), methodology (equal), data curation (equal), visualization (equal), writing – original draft (supporting), writing – review and editing (equal); B. F. E. C. conceptualization (supporting), methodology (equal), writing – original draft (supporting), writing – review and editing (equal); D. A. conceptualization (supporting), methodology (equal), data curation (equal), writing – original draft (supporting), writing – review and editing (equal).

## Conflicts of interest

There are no conflicts to declare.

## Data Availability

The PCE prototype is available online at https://photochem-explorer.com. The studies used in developing the prototype are drawn from ref. 45 and 68–79 and their metadata is available on the platform.

## Appendices

### Implementation of the PCE prototype

The web frontend of the prototype is implemented using React 18 with TypeScript and styled using Tailwind CSS, the shadcn/ui component library, and Lucide icons. The backend is implemented in Python 3.13 using FastAPI for routing and Uvicorn as the ASGI server. Molecule structures are processed using RDKit^57^ for SMILES-to-SMARTS conversion and Accurate Functional Group Extraction and Molecular Structure Comparison (AccFG^58^) for functional group detection, and enriched with metadata retrieved from the PubChem API^59^ using PubChemPy.^60^ Publication metadata is retrieved through the CrossRef API^61^ and DOI.org.^62^ To visualize molecules, we use 3Dmol.js^63^ together with PubChem and ETKDGv3 (ref. 64) as 3D fallback, and SmilesDrawer^65^ as a 2D fallback. Currently, users can optionally link data repositories to a publication. Links pointing to Zenodo records are validated and display a preview of the record metadata (title, creators, and files). Data validation occurs within both layers: on the frontend using Zod schemas with react-hook-form, and on the backend using Pydantic models. Database access uses SQLAlchemy ORM with parameterized queries. Access control is enforced *via* JSON Web Tokens (JWT) with role-based permissions for users, moderators, and administrators. The system is deployed on Google Cloud Platform (GCP) using Cloud Run. Persistent data is stored in a managed PostgreSQL 17 database provided through GCP Cloud SQL. AI-assisted coding tools, including Cursor^66^ and Claude,^67^ were used during software development and code refactoring.
